# COVID-19 teleassistance and teleconsultation: a matched case-control study (MIRATO project, Lombardy, Italy)

**DOI:** 10.3389/fcvm.2023.1062232

**Published:** 2023-08-14

**Authors:** Palmira Bernocchi, Giacomo Crotti, Elvira Beato, Francesco Bonometti, Vittorio Giudici, Patrizia Bertolaia, Elisa Perger, Andrea Remuzzi, Tiziana Bachetti, Maria Teresa La Rovere, Laura Adelaide Dalla Vecchia, Fabio Angeli, Gianfranco Parati, Gabriella Borghi, Michele Vitacca, Simonetta Scalvini

**Affiliations:** ^1^ Continuity Care and Telemedicine Service, Istituti Clinici Scientifici Maugeri IRCCS, Institute of Lumezzane, Brescia, Italy; ^2^ Epidemiology Unit, Bergamo Health Protection Agency, Bergamo, Italy; ^3^ Department of Cardiac Rehabilitation, Bolognini Hospital, Azienda Socio Sanitaria Territoriale Bergamo Est, Bergamo, Italy; ^4^ Socio-Health Management Direction, Azienda Socio Sanitaria Territoriale Bergamo Est, Bergamo, Italy; ^5^ Istituto Auxologico Italiano, IRCCS, Sleep Disorders Center & Department of Cardiovascular, Neural and Metabolic Sciences, San Luca Hospital, Milan, Italy; ^6^ Department of Management, Information and Production Engineering, University of Bergamo, Bergamo, Italy; ^7^ Scientific Direction, Istituti Clinici Scientifici Maugeri IRCCS, Pavia, Italy; ^8^ Cardiac Rehabilitation Division, Istituti Clinici Scientifici Maugeri IRCCS, Institute of Montescano, Pavia, Italy; ^9^ Department of Cardiology, Istituti Clinici Scientifici Maugeri IRCCS, Institute of Milan, Milan, Italy; ^10^ Department of Medicine and Technological Innovativon (DiMIT), University of Insubria, Varese, Italy; ^11^ Department of Medicine and Cardiopulmonary Rehabilitation, Istituti Clinici Scientifici Maugeri IRCCS, Institute of Tradate, Varese, Italy; ^12^ Department of Cardiology, Istituto Auxologico Italiano, IRCCS, San Luca Hospital, Milan, Italy; ^13^ Department of Medicine and Surgery, University of Milano-Bicocca, Milan, Italy; ^14^ Department of Respiratory Rehabilitation, Istituti Clinici Scientifici Maugeri IRCCS, Institute of Lumezzane, Brescia, Italy; ^15^ Department of Cardiac Rehabilitation, Istituti Clinici Scientifici Maugeri IRCCS, Institute of Lumezzane, Brescia, Italy

**Keywords:** teleassistance, telemonitoring, teleconsultation, telemedicine, chronic disease, rehabilitation, outcome, symptoms

## Abstract

**Background:**

During the COVID-19 pandemic, telemedicine has been recognised as a powerful modality to shorten the length of hospital stay and to free up beds for the sicker patients. Lombardy, and in particular the areas of Bergamo, Brescia, and Milan, was one of the regions in Europe most hit by the COVID-19 pandemic. The primary aim of the MIRATO project was to compare the incidence of severe events (hospital readmissions and mortality) in the first three months after discharge between COVID-19 patients followed by a Home-Based Teleassistance and Teleconsultation (HBTT group) program and those discharged home without Telemedicine support (non-HBTT group).

**Methods:**

The study was designed as a matched case-control study. The non-HBTT patients were matched with the HBTT patients for sex, age, presence of COVID-19 pneumonia and number of comorbidities. After discharge, the HBTT group underwent a telecare nursing and specialist teleconsultation program at home for three months, including monitoring of vital signs and symptoms. Further, in this group we analysed clinical data, patients' satisfaction with the program, and quality of life.

**Results:**

Four hundred twenty-two patients per group were identified for comparison. The median age in both groups was 70 ± 11 years (62% males). One or more comorbidities were present in 86% of the HBTT patients and 89% in the non-HBTT group (*p* = ns). The total number of severe events was 17 (14 hospitalizations and 3 deaths) in the HBTT group and 40 (26 hospitalizations and 16 deaths) in the non-HBTT group (*p* = 0.0007). The risk of hospital readmission or death after hospital discharge was significantly lower in HBTT patients (Log-rank Test *p* = 0.0002). In the HBTT group, during the 3-month follow-up, 5,355 teleassistance contacts (13 ± 4 per patient) were performed. The number of patients with one or more symptoms declined significantly: from 338 (78%) to 183 (45%) (*p* < 0.00001). Both the physical (ΔPCS12: 5.9 ± 11.4) component and the mental (ΔMCS12: 4.4 ± 12.7) component of SF-12 improved significantly (*p* < 0.0001). Patient satisfaction with the program was very high in all participants.

**Conclusions:**

Compared to usual care, an HBTT program can reduce severe events (hospital admissions/mortality) at 3-months from discharge and improve symptoms and quality of life.

**Clinical trial registration:**

www.ClinicalTrials.gov, NCT04898179.

## Introduction

The rapid spread of disease and the increase in confirmed cases of acute respiratory infection due to the SARS-CoV-2 has posed an enormous challenge to healthcare systems worldwide (1).

Telemedicine proved an invaluable way to provide access to care when social distancing created a barrier to face-to-face care. Some medical centres responded to COVID-19 by rapidly adopting telemedicine and virtual care (2).

In particular, patients with chronic conditions, such as cardiovascular disease (3–5), diabetes (5, 6), chronic respiratory disease (7), and other comorbidities, for whom it was impossible to have ambulatory visits, obtained benefits from telemedicine in terms of usual follow-up leading to a reduction of acute exacerbations and improvement in quality of life.

Furthermore, during the COVID-19 pandemic, telemedicine has been recognized as a powerful modality to shorten the length of hospital stay (8) and to free up beds for the most seriously ill patients, but also—by monitoring patients outside of the care setting (4)—to reduce re-access to the Emergency room and hospital and reduce deaths (9–11).

Indeed, the patients discharged after a COVID-19 hospitalization have a high rate of readmission and death due to the high number of complications (10) and the numerous comorbidities often present (12, 13).

Lombardy, particularly the Bergamo, Brescia and Milan areas, was one of the regions in Europe most hit by the COVID-19 pandemic (1, 14, 15), both during the first wave from March to May 2020 and the second wave from August 2020 through May 2021 (16).

The research problems were the need to quickly make hospital beds available given the large number of Covid-19 patients requiring hospitalization and the need to discharge patients hospitalized for severe COVID-19, not fully recovered.

Based on the experience of our hospitals where telemedicine and teleassistance (17) for chronic patients (18, 19) was common practice, the research question to address was the clinical relevance of the adoption of telemedicine in the treatment of patients in the early phase following a COVID-19 hospitalization.

The primary aim of the MIRATO project was to compare the incidence of severe events (hospital readmissions and mortality) in the first three months after discharge between COVID-19 patients followed by a Home-Based Teleassistance and Teleconsultation (HBTT group) program and those discharged home without Telemedicine support (non-HBTT group).

The secondary aims concerned only the HBTT group and analysed: (i) the home-based telecare and teleconsultation activity during the follow-up period and the data obtained with specific instrumental assessments; (ii) the improvement of the patient's clinical symptoms and the disorders related to emotional and mood status; (iii) the patients' satisfaction with the program and the quality of life changes (SF 12 instrument).

## Materials and methods

### Study design and participants

The study was designed as a matched case-control study.

The study participants were divided into two groups: (1) the HBTT group and (2) the non-HBTT group, which served as a comparison group.

### HBTT group

Four hospitals in Lombardy with previous experience in telemedicine programs [(Istituti Clinici Scientifici IRCCS Maugeri, Centres of Lumezzane (Brescia) and Tradate (Varese); Bolognini Hospital Seriate (Bergamo); and IRCCS Istituto Auxologico Italiano Hospital (Milano)] were involved to enrol patients in HBTT group.

Consecutive consented patients were eligible for the program if they had been hospitalized for COVID-19 infection.

The exclusion criteria were age <18 years, patient refusal, or patient transfer to other facilities upon discharge (e.g., rest home, other hospitals).

### HBTT program

A tested telemedicine program was used for COVID-19 patients and extensively described previously (17, 20).

Patients in the HBTT group were followed through scheduled telephone calls or video consultations. A Nurse case manager was assigned to each patient, whose role was to improve the patient's and family's awareness of the disease and to support them in the management, reduce anxiety associated with the presence of symptoms and improve the patient's lifestyle through counselling.

During each contact, the nurse performed a standardized interview, including the following information:

- Body temperature, heart rate, systolic/diastolic blood pressure, oxygen saturation
- General condition: asthenia, dysgeusia, anosmia, headache, myalgia
- Cardiovascular condition: palpitations, chest pain, peripheral oedema
- Respiratory symptoms: cough, dyspnoea, sputum
- Gastrointestinal symptoms: nausea/vomiting, diarrhoea
- Other clinical information
- Emotional status: nervous, tense, worried, sad, crying more than usual, difficulty in relationships, no interest in things, needs help, confident about the future.

The nursing activities in particular included:

- response to requests for assistance regarding late symptoms related to COVID-19 and the other comorbidities
- management of any clinical data transmitted electronically
- support to ensure adherence to medical and personalized physical therapy prescribed at discharge
- help patients to restart their previous long-term therapy or find the proper dosage of drugs (following the specialist's prescription)
- to increase patient empowerment in the management of comorbidities
- filling in electronic medical records for consultation by the referring clinicians.

During the first two weeks after discharge, the nurse made contact daily; in the following two weeks, contacts were reduced to one call per week (or more if the patient needed it). During the last two months, the nurse made contact only if the patient required it. In case of symptoms or problems, the patient could call the service. In any case, it did not replace the emergency service. Finally, at the end of the third month, the nurse contacted the patients to check their clinical condition and to close the program.

For the home program, all patients were required to have a pulse oximeter to measure oxygen saturation in the blood. If they did not have one, it was provided.

A portable single-lead ECG was also provided in selected cases to detect any arrhythmias.

An oxygen saturation detection system was provided for patients still on oxygen therapy when discharged, consisting of a digital trend oximeter (O2Ring ™ Continuous Ring Oxygen Monitor, Wellue®) with Bluetooth transmission and a dedicated smartphone. The communication between devices via Bluetooth provided an uninterrupted flow of data, usable for continuously monitoring the patient's vital parameters and viewable on the telemedicine platform (TelMed, HTN, Brescia, Italy).

At the start of the program, one week after hospital discharge, and at the end, the validated Italian version of the SF-12 quality of life questionnaire was administered to patients during the phone calls. The scores of the quality-of-life domains range from 0 to 100, with higher scores indicating higher quality of life. The two summary scores on quality of life domains were collected and analysed following published indications (21, 22). A satisfaction questionnaire investigating the program's usefulness, clarity of the indications and satisfaction with the answers provided by the nurses was administered at the end of the program.

A COVID-19 monitoring electronic sheet (20) was used to record any parameter and emotional status referred to during the contact by the patient. Based on the patient's answers, the nurse was instructed to capture indicators of instability, requiring the psychologist's advice.

### Non-HBTT group

Three Istituti Clinici Scientifici Maugeri hospitals with no available telemedicine services (Pavia, Milano and Montescano Centers) collected data on patients hospitalized for COVID-19 at discharge and after three months. The information on events, rehospitalizations or deaths, was investigated by consulting outpatient visits, if present, or contacting the patients or their relatives by phone.

### Data collected and analysed in both groups

At baseline, age, sex, COVID-19 infection diagnosis, presence of interstitial pneumonia, need for ventilation, and number and type of comorbidities were recorded. At three months from discharge, events (hospital readmission and mortality) for all causes were recorded.

### Ethics and consent to participate

All interventions were carried out by the Declaration of Helsinki, and the study was approved by the Ethics committees of the centres involved (ICS Maugeri on June 30, 2020, San Luca Auxologic Hospital on September 29, 2020 and ASST Bergamo Est, Bolognini Hospital on October 19, 2020). The Project was registered at Clinical trial.gov NCT04898179). All participants gave informed consent. The analysed data were completely anonymized.

### Data analysis

#### Pairing 1:1 case: control

To have a suitable group of controls for cases (23), we selected from the control file those subjects who matched with the cases by the following variables: age in five-year classes, sex (male or female with no other modification), presence of COVID SARS pneumonia and number of comorbidities aggregated in the following categories: 0–1 comorbidities or 2–3 comorbidities or 4–6 comorbidities or ≥7 comorbidities).

### Statistical analysis

Continuous variables were expressed as mean ± standard deviation (SD), and categorical variables as number and percentage. Statistical analyses were performed using Graph Pad Prism 8. Differences in baseline characteristics between groups were examined using parametric (*t*-test) and non-parametric (chi-square) tests.

The comparison (primary outcome) of the combined event (hospital readmission and mortality) between the case group and control group was performed by survival analysis (Kaplan–Meier) of time to the first event in 3 months, whereas the time to first event difference between groups was evaluated with the Log-rank test. The primary outcome of the Kaplan–Meier analysis is the hazard ratio (HR) which expresses the magnitude of the difference between control and case groups with the *p* value and 95% confidence interval measuring the statistical significance of the difference.

The c-square analysis was performed to compare frequencies of problems/symptoms observed between the start and end of the home-based telecare period. A paired *t*-test for two samples with equal variance on the two SF-12 components (PCS and MCS) to determine a significant pre-post difference. The alpha level for all analyses was set at 0.05.

## Results

From July 2020 to September 2021, 510 patients underwent the HBTT program. Data on 728 non-HBTT patients were collected retrospectively and matched by age, comorbidity class, sex, and presence of COVID-19 pneumonia with each subject in the HBTT group.

Following the matching, we obtained two groups of 422 (83% of the original 510) HBTT cases and 422 non-HBTT controls (58% of the original 728).

Baseline characteristics of two groups are presented in Table 1.

**Table 1:** Baseline characteristics of HBTT and non-HBTT patients.

	HBTT Case group *N* = 422	Non-HBTT Control group *N* = 422	*p*
M/F (%)	262/160 (62%/38%)	262/160 (62%/38%)	1
Mean age ± SD (years)	70 ± 11	70 ± 11	0.9616
Females, mean age ± SD (years)	72 ± 11	72 ± 11	0.9691
Males, mean age ± SD (years)	69 ± 12	69 ± 12	0.9747
Acute phase			
Pneumonia from COVID-19 (%)	422 (100%)	422 (100%)	1
Patients needing mechanical Ventilation	170 (40%)	165 (39%)	0.7784
Invasive ventilation	53 (12.5%)	37 (9%)	0.0944
Principal comorbidities (%)			
Diabetes	85 (20%)	97 (23%)	0.3572
Hypertension	243 (57%)	216 (51%)	0.0724
Hyperlipidemia	79 (19%)	63 (15%)	0.1675
Obesity	65 (15%)	53 (13%)	0.2749
Chronic renal failure	36 (8%)	40 (9.5%)	0.7183
Chronic heart disease	100 (24%)	107 (25%)	0.6312
Chronic respiratory failure	88 (21%)	85 (20%)	0.8646
Neurological disease	30 (7%)	39 (9%)	0.3149
Number of comorbidities/patients, mean ± SD	2.8 ± 2.0	2.9 ± 2.0	0.4679
Patients with 0 comorbidities	57 (14%)	45 (11%)	0.2454
Patients with 1–2 comorbidities	152 (36%)	146 (34%)	0.6654
Patients with ≥3 comorbidities	213 (51%)	232 (55%)	0.2146

SD, standard deviation.

### Primary aim

The total number of severe events occurring in the two groups was 59, 17 (14 hospitalizations and 3 deaths) in the HBTT group and 42 (26 hospitalizations and 16 deaths) in the non-HBTT control group (*p* < 0.001). Pooling the data together, the causes of the first events were respiratory in 42% of the cases, cardio-vascular in 24% and other causes in 34%.

**Figure 1:**
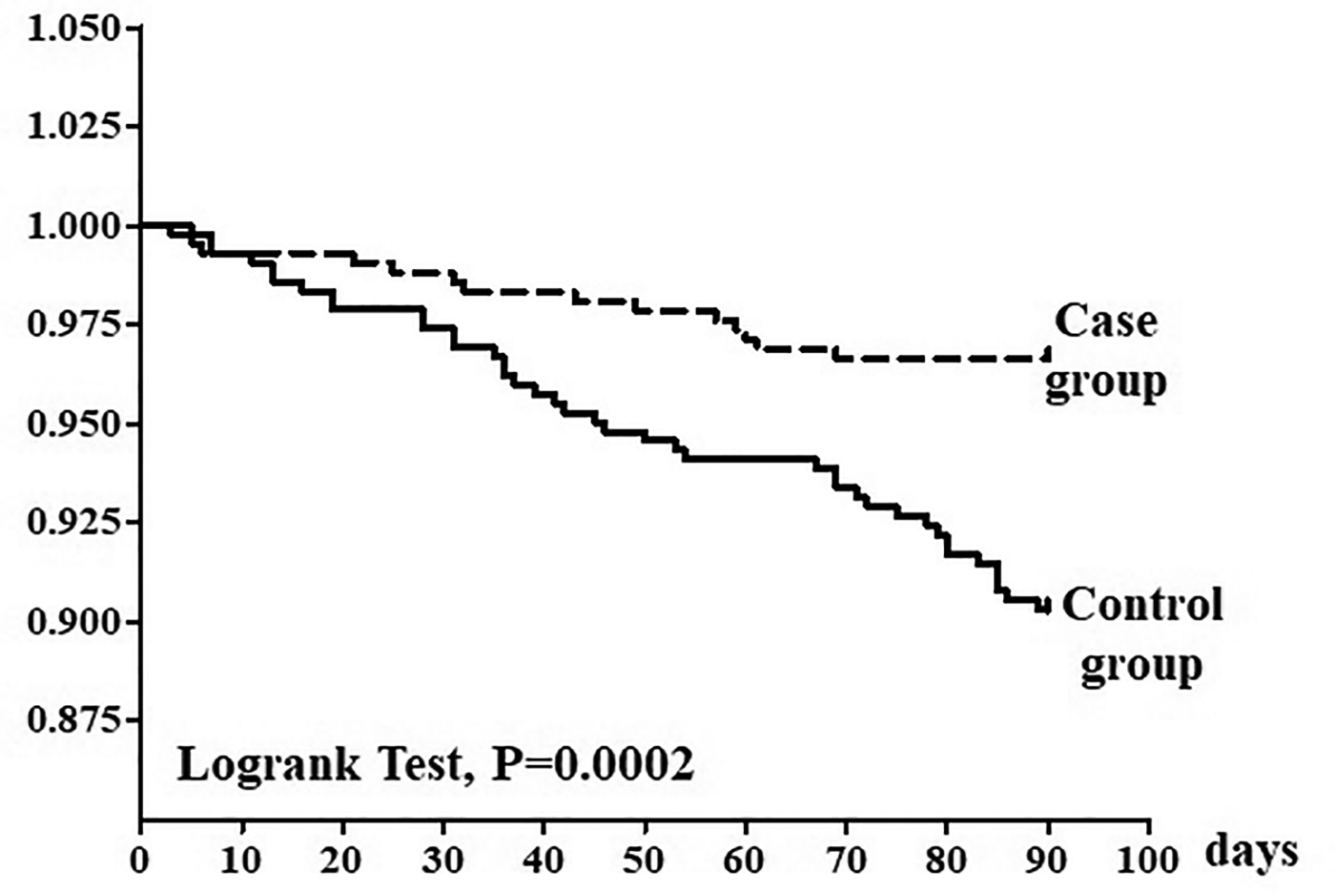
Kaplan–Meier survival analysis of time to first combined event (hospital readmission and mortality). Survival difference between groups was evaluated with the log rank test. The solid line represents the control group and dotted line represents the intervention group.

Considering combined data of hospitalizations and deaths, Figure 1 shows the Kaplan–Meier survival curve (HR = 2.955, 95% confidence interval: 1.589 - 4.575; Log-rank test *p* < .001).

### Secondary aims (only in HBTT case group)

i. *Home-based telecare and teleconsultation activity during the follow-up period*.

During the study period, in the HBTT group, 5,355 phone contacts and 25 videoconferences took place, a mean of 13 ± 4 contacts per patient: 5,308 were initiated by nurses, and 72 were requested by patients. Of the nurse actions, 90% (5,191) focused on counselling, checking medical and physical therapy, reinforcing educational programs and checking patients’ clinical data. In 10% of the contacts, the nurse required teleconsultation with a specialist. Of the 322 teleconsultations with a specialist (cardiologist, pneumologist, internist, psychologist), 77% were related to therapy changes and 23% to some clinical problems.

At the end of the HBTT program, 367 patients closed the path through a telephone contact with the nurse, 19 with a tele-visit and 33 with an outpatient visit.

In the subgroup of patients (*n* = 29, 66 ± 12 years) discharged with oxygen support for severe dyspnoea on mild or moderate effort and provided with a continuous digital oximeter, 440 saturation times were recorded: 123 at night, 148 during physical activity and 173 at rest. The intervention of the pulmonologist for dyspnoea (in 3 cases) and desaturation (in 2 cases) during physical activity was activated by teleconsultation.

ii. *Improvement of the patient's clinical symptoms and the disorders related to emotional and mood status*.

**Figure 2:**
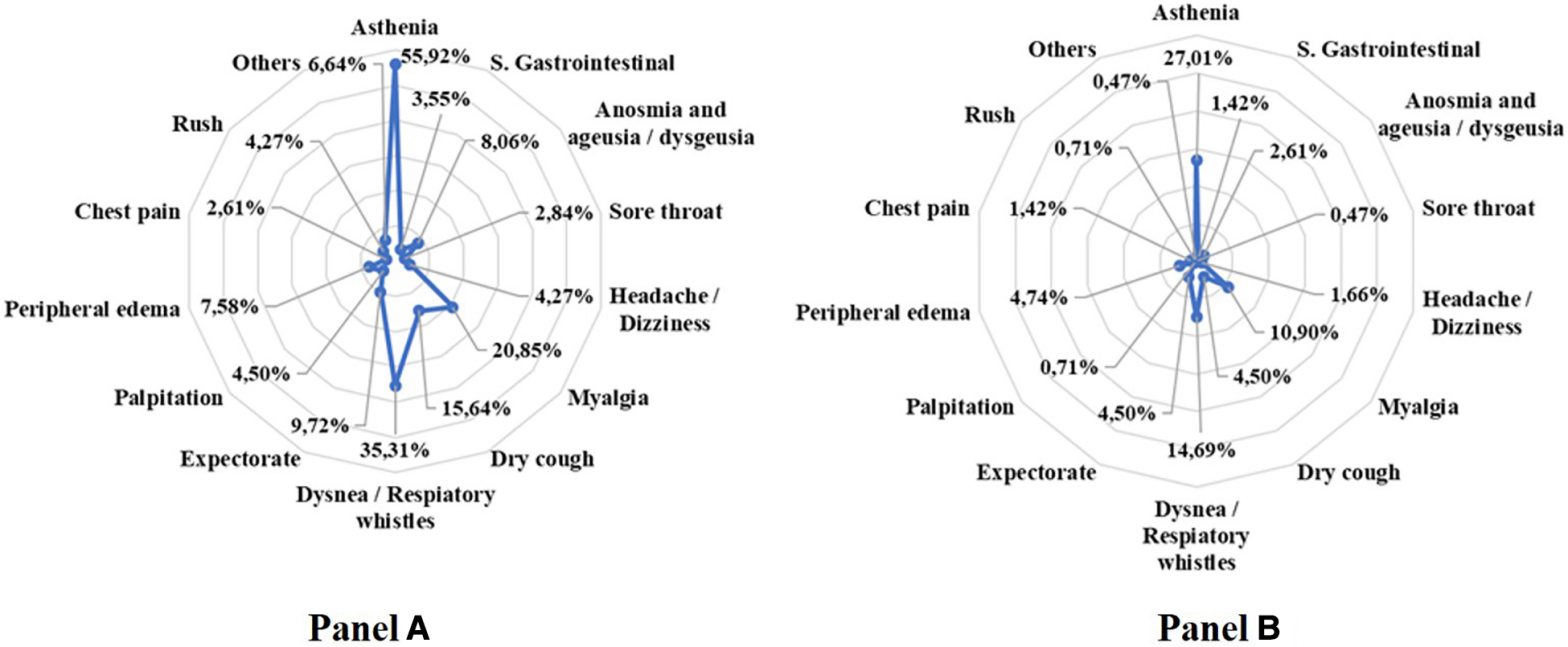
Patient symptoms presentation at the beginning (Pane A) of the HBTT program and at the end of 3 month (Panel B).

Changes in clinical symptoms are represented in Figure 2. At the beginning of the HBTT program, 338 (78%) patients complained of one or more symptoms (panel A) compared to 183 (45%) patients at the end (panel B), *p* < 0.001.

Of the 422 patients, 152 (36%) showed emotional disorders detected by the nurse during their home journey. In 95% of cases, these disorders were solved with the support of the nurse and caregivers. However, for 20 (5%) patients, psychological support was needed.

In Table 2, we show the changes for the different items of patient's emotional status detected by the nurse at the beginning and end of the program.

**Table 2:** Patients’ emotional status at the beginning of the program and after 3 months.

Emotional state	At the beginning (*N* = 152 patients)	After 3 months (*N* = 152 patients)	*p*
Nervous, tense, and worried	85 (20%)	38 (9%)	<0.001
Sad	72 (17%)	33 (8%)	0.001
Crying more than usual	42 (10%)	16 (4%)	0.001
Difficulty in relationships	31 (7%)	17 (4%)	ns
No interest in things	28 (7%)	17 (4%)	ns
Needs help	79 (19%)	37 (9%)	<0.001

However, 267 patients (63%) felt confident in the future at the beginning of the program, rising to 355 (85%) at the end (*p* < 0.001).

iii. *Patients' satisfaction with the program and quality of life (SF 12 instrument)*.

Table 3 reports the results of the SF-12 quality of life questionnaire. Both the physical component of SF-12 and the mental components of SF-12 improved significantly at the end of the 3-month program, *p* < 0.001).

**Table 3:** SF-12 quality of life questionnaire at the beginning and end of the home-based telecare period**.**

SF-12	T0 (*N* = 360)	T1 (*N* = 336)	Delta (t1-t0) (*N* = 336)	*p*
PCS12	39.4 ± 8.2	45.3 ± 9.8	5.9 ± 11.4	<0.001
MCS12	46.3 ± 11.3	51.0 ± 11	4.4 ± 12.7	<0.001

PCS, physical component summary; MCS, mental component summary.

Patient satisfaction with the program was very high in all evaluated patients, with an overall mean score of 7.7 ± 0.8.

## Discussion

Based on the feasibility and effectiveness of telemedicine in the follow-up of patients with chronic diseases (24–26), also confirmed for those who could not access their routine ambulatory visits during the COVID-19 pandemic (26–30), the MIRATO Project demonstrated that COVID-19 patients followed by a Home-Based teleassistance and teleconsultation program had a significantly lower rate of hospitalizations and mortality compared to the non-HBTT control group.

Our results align with Patel et al. (8), who showed that home telemonitoring after discharge for patients with COVID-19 is a safe tool that may reduce the mean duration of hospitalization and create more bed capacity.

Steinberg et al. (31), in a remote monitoring program for patients with COVID-19, observed a reduction in emergency room and hospital and Intensive Care Unit admissions. As differences in baseline health status could have confounded the observed results, the authors (31) concluded that a randomized clinical trial would provide more conclusive evidence on the effect of remote monitoring device utilization. Given the difficulty of randomizing, we decided to use a non-randomized case-control study. Our study showed fewer hospitalizations and deaths in the HBTT patients compared to the non-HBTT patients. A likely explanation of our results is that the nurse's early recognition of a critical (unstable) condition made it possible to provide a therapeutic intervention effective in avoiding subsequent hospitalization and death.

Other important results were to keep the evolution of the disease under control, provide counselling and support to patients, assist recovery, and identify and solve the first signs and symptoms related to a possible recurrence of COVID-19 disease and comorbidities. Through Teleassistance, nurses tried to help patients manage their symptoms and the daily problems encountered and to reduce the sense of isolation generated by the lockdown, which had a positive, albeit partial, effect on the patient's state of anxiety. Several factors undoubtedly contributed to determining this anxiety and depression or worsening it if already present. As reported in the recent literature on COVID-19 (32, 33), telepsychology support should be part of a proactive governance model to ensure the continuity of mental health care services.

In the MIRATO Project, telepsychology was an important component, even if reserved only for the most severe situations. This support meant patients could also report disorders related to their emotional status and mood during contact with the nurse. In some cases, it was possible to activate the psychological support.

As described by Gazzi et al. (34), this home-based approach centred on the role of the nursing case manager not only determined the improvement of the patient's health state but also an improvement in the patient's moving picture and the caregiver feels supported during the post phase recovery. In this paper, the authors described good acceptability and satisfaction regarding a tele-psychological support service provided “on-demand” for patients, and their caregivers, followed by a case manager nurse with a telehealth and telecare service.

Some recent publications supported that telenursing could have the capacity to facilitate the care and support of a large number of patients and that this home-based approach helps patients and caregivers to reduce anxiety (35–38).

A digital continuous-detection system of oxygen saturation was provided to patients still on oxygen therapy when discharged, permitting real-time intervention in the case of need.

As the literature reports, telemedicine promotes patient empowerment and enables healthcare professionals to make more informed and accurate decisions. It is crucial to help patients, particularly chronic patients, to take responsibility for their health status and self-care (39). It was imperative for COVID-19 patients, given their difficulty coming to the hospital for organized follow-ups during a pandemic.

Previous experience with the Maugeri Telehealth system (17) has run for approximately 20 years; this long and consolidated experience has paved the way to rapidly set up a telemedicine-based program for managing COVID-19 patients (20). It was essential given the frailty of the COVID-19 patients. We recorded the Nurse case manager's activity focused on counselling, verification of medical therapy, educational reinforcement, and reporting of patients' clinical data in a computerized clinical file. The result of this activity was an improvement in patients' physical and mental health, as indicated in the SF-12 questionnaire on quality of life.

## Study limitations

A notable limitation of this research includes the retrospective nature of the study and the impossibility of planning a randomized controlled study which was not justified during the COVID-19 pandemic. Another limitation was the partial extraction of data from the electronic medical record, which may have sometimes been only partially accurate.

## Conclusions

Compared to the control group, a significant reduction in composite events (hospital admissions and mortality) was found in the HBTT group. Due to the impossibility of running a randomised control study, this result might be overestimated, but it remains impressive given the current critical situation. These findings demonstrate the feasibility of recruiting more COVID-19 patients from different hospitals, even in various geographical areas. It could be possible to apply telemedicine service even in a post-acute phase and not only in a chronic phase of the disease. Future studies should be focused on these post-acute patients and on the possibility of applying different methodologies for the follow-up of chronic patients as the hybrid model (both remote and face-to-face visits).

## Data Availability

The raw data supporting the conclusions of this article will be made available by the authors, without undue reservation.

## MIRATO study group

Istituti Clinici Scientifici Maugeri IRCCS, Lumezzane, Brescia, Italy: Irene Taccolini, Isa Serlini, Giuliano Assoni, Margherita Zanardini, Simona Botelli, Elisa Pasotti, Michele Vitacca: Istituti Clinici Scientifici Maugeri IRCCS, Tradate, Italy: Antonella Rimoldi, Mingardi Samantha Chiara: Bolognini Hospital, Azienda Socio Sanitaria Territoriale Bergamo Est, Seriate, Bergamo, Italy: Paola Neri, Patrizia Rocca, Luigina Viscardi, Pagani Ilaria, Elisabetta Santoro: San Luca Hospital, IRCCS Istituto Auxologico Italiano, Milan, 20149, Italy: Debora Rosa, Antonina Orlando: Bergamo Health Protection Agency, Bergamo, Italy: Carlo Tersalvi, Alberto Zucchi, Roberta Ciampichini, Giulia Baroni: University of Bergamo, Italy: Caterina Rizzi, Andrea Vitali, Anna Ghidotti, Anna Savoldelli: Istituti Clinici Scientifici Maugeri IRCCS, Pavia, Italy: Antonella Balestrino, Francesco Fanfulla, Piero Ceriana, Luca Chiovato: Istituti Clinici Scientifici Maugeri IRCCS, Montescano, Italy: Angelo Caporotondi, Giampaolo Guazzotti, Marta Lovagnini: Istituti Clinici Scientifici Maugeri IRCCS, Milano, Italy: De Maria Beatrice, Sideri Riccardo, Maurizio Bussotti.
